# To Nutraceuticals and Back: Rethinking a Concept

**DOI:** 10.3390/foods6090074

**Published:** 2017-09-05

**Authors:** Antonello Santini, Ettore Novellino

**Affiliations:** Department of Pharmacy, University of Napoli Federico II, Via D. Montesano, 49-80131 Napoli, Italy; ettore.novellino@unina.it

The concept of nutraceuticals as pharma-foods comes from far. This term is made from the two words “nutrient” and “pharmaceutical”, was coined by Stephen DeFelice, and is defined as “a food or part of a food that provide medical or health benefits, including the prevention and/or treatment of a disease” [1].

This definition leads to a partial overlap with the definition of a food supplement. In fact, both claim beneficial effects for health; however, while nutraceuticals are made from food or part of a food, food supplements are single substances used alone or in mixtures with the scope of adding micronutrients when the body is in need of them. 

The aspect outlined by DeFelice [1]—in particular the preventive aspect and the treatment of a disease—is absent in the definition and scope of food supplements, which can be an aid for the body but are not required to have a proven clinical efficacy on a health condition. Based on these considerations, it hence appears of utmost importance to develop a new definition for nutraceuticals foreseeing their use “beyond the diet, before the drugs” as tools which can be able to prevent or delay the onset of some asymptomatic long term pathological conditions (e.g. hypercholesterolemia, hypertriglyceridemia, etc.). The steps involved in a new nutraceutical formulation should start with the identification of the target pathologic condition, in a way similar to what happens for drugs.

Figure 1 shows the steps to take when assessing the possible use of a nutraceutical. It is of utmost importance the clinical target identification and the appropriate food matrix to use. The safety and the in vitro and in vivo tests are crucial. The differences between nutraceuticals and food supplements (e.g. mineral or protein food supplements) are also outlined, stressing the necessity of clinical evidences substantiating the health efficacy for nutraceuticals based on safety, efficacy, and known mechanism of action.

Once a pathologic health condition target has been identified, formulation can be prepared from vegetal or animal matrices, and tested in vitro and in vivo, keeping in mind that safety and efficacy must be substantiated by clinical tests [2].

Nutraceuticals which are extracted from vegetable sources (phytocomplex) or which are the active metabolite complex (if of animal origin) should hence be understood as a set of pharmacologically active substances which have inherent therapeutic properties due to the natural active principles of recognized effectiveness which they contain. They should be administered in the appropriate pharmaceutical form (e.g., capsule, tablet, drink, etc.). Incidentally, these forms of administration coincide with those used for both drugs and food supplements.

The assessment of nutraceuticals’ optimal conditions of use should be complementary with safety information as well as bioavailability and bioaccessibility information, so that they can propose themselves as a powerful toolbox to be used to prevent and cure some pathologic conditions in subjects who, for example, are not eligible for conventional pharmacological therapy. 

For this reason, and due to their natural origin, a growing demand exists for nutraceuticals, which shade the frontier existing between pharmaceuticals and food, and this is also helping the producers to diversify their agriculture and promote research and innovation. Nonetheless, different country-specific regulations, safety, and health claim substantiation are the main challenges which the nutraceuticals are experiencing. The main challenge is the absence of a shared supra-national regulation for nutraceuticals, which would recognize their potential and possible role as therapeutic tools in some pathological conditions based on assessed safety, known mechanism of action, clinically proven efficacy in both reducing the risk of illness onset and enhancing overall well-being. 

The labelling of marketed products is another source of confusion, and is often due to misinformation, which could induce false expectations regarding beneficial health effect and miss the target for a product to be effective as claimed. What may be considered a functional food under a given set of circumstances may be deemed a dietary supplement, medical food, food for special dietary use, a nutraceutical, or a drug under different circumstances, depending on its ingredients and the claims as reported on the label [3].

While the definition of food supplement is quite clear and understandable (see Table 1), the definition of nutraceutical still lies in between food, food supplement, and pharmaceutical, and the legitimate assessment of their potential in medicine is still contradictory and far from being shared and accepted worldwide [4].

The definition of a food supplement often overlaps with the one accepted for nutraceuticals as present in the collective imagination, and the rationale behind their use is becoming a challenge of this millennium [8,9]. 

Food supplements should be, as per their micronutrients content, addressed to improve health if appropriately targeted to those in need. Nevertheless, many of the health claims which are currently associated to food supplements, pro- and pre-biotics, as well as herbal products and functional foods are often not properly substantiated by in vivo data on safety, efficacy, and effect on health and/or on pathologic conditions. This is mainly due to the lack of in vivo and mechanism of action studies confirming the claimed health beneficial effect. Many literature data refer to in vitro studies, and focus on single food constituents (micronutrients). Any health beneficial effect for nutraceuticals is related to the fact that they derive from food or part of food, and consequently they can be considered safe or generally recognized as safe (GRAS). Safety is of utmost importance, since possible contaminants of inorganic [10] and organic origin [11] can contaminate these products and cause health issues. 

It seems necessary to restructure the entire regulatory framework of dietary supplements and include nutraceuticals as a new category, by giving credit to their role in the prevention and cure of some pathological conditions. The pre-market approval system should be under any circumstance substantiated by in vivo clinical data to determine and assess their safety and efficacy. This approach could look similar to the one used for pharmaceuticals, which includes clinical trials to in vitro and safety tests. The likelihood of this happening in the foreseeable future is unfortunately quite low, but it seems reasonable to hypothesize that the competent national authorities could ask the manufacturers to provide data that substantiates safety, efficacy, and mechanism of action of any claims attributed to food supplements and nutraceuticals, avoiding any possible source of confusion. 

## Figures and Tables

**Figure 1:**
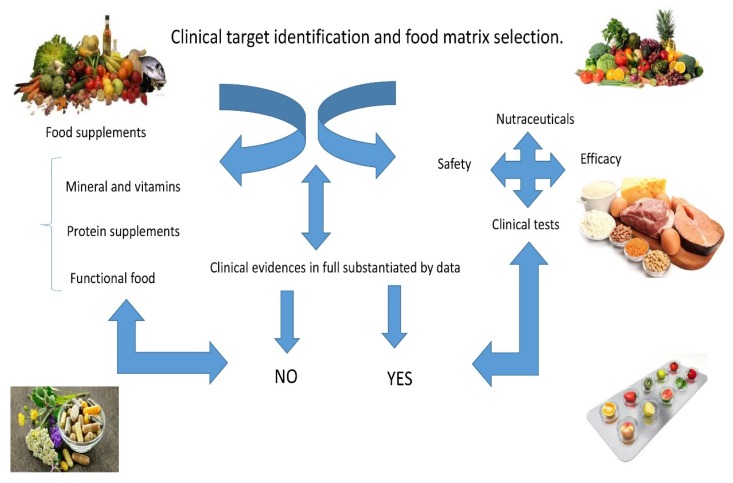
Food supplements and nutraceuticals.

**Table 1:** Some definitions.

Terms	Definitions	Reference
Food supplement	A product (other than tobacco) in the form of a capsule, powder, softgel, or gelcap intended to supplement the diet to enhance health that bears or contains one or more of the following dietary ingredients: a vitamin, mineral, amino acid, or other botanical or dietary substance.	United States Government Office, 1994 [5]
Nutraceuticals	Food or part of food that provides medical or health benefits, including the prevention and/or treatment of a disease.	De Felice, 1995 [1]
Nutraceuticals	Nutritional products that provide health and medical benefits, including the prevention and treatment of disease.	European Nutraceutical Association, 2016 [6]
Functional food	Any food or ingredient that has a positive impact on an individual’s health, physical performance, or state of mind, in addition to its nutritive value.	Hardy, 2000 [7]
